# Listening comprehension and its influence on reading fluency in primary students with special educational needs: a study in mainstream inclusive classrooms

**DOI:** 10.3389/fpsyg.2026.1678170

**Published:** 2026-02-03

**Authors:** Shuting Zhang, Dengfeng Ren, Jiaojiao Wu

**Affiliations:** 1 The Faculty of Education, Southwest University, Chongqing, China; 2 School of Teachers, Guizhou University of Engineering Science, Bijie, China

**Keywords:** inclusive education, listening comprehension, primary students, reading fluency, special educational needs

## Abstract

Developing literacy is a fundamental goal of public education. In pursuit of inclusive and equitable quality education (SDG 4), a deeper understanding of literacy development in pupils with special educational needs (SEN) is essential. Guided by the Direct and Indirect Effects Model of Reading (DIER), this longitudinal study investigated the development of listening comprehension (LC) and its role in reading fluency among a heterogeneous sample of SEN pupils (*N* = 103) identified by teachers in Chinese inclusive primary schools. Standardized assessments showed that pupils with SEN had lower LC than their typically developing peers in the same grades, and this gap widened over 1 year. Robust regression analysis revealed that vocabulary exerted a substantial positive effect on LC, while gender and grade were not. Both LC and orthographic knowledge significantly predicted reading fluency. These findings highlight the potential benefits of (1) targeted interventions to strengthen vocabulary, LC, and orthographic knowledge in pupils with SEN, and (2) refining teacher-based SEN identification procedures, which may contribute to enhancing inclusive education quality and promoting educational equity.

## 1 Introduction

The United Nations’ 2030 Agenda for Sustainable Development explicitly identifies the goal of “ensuring inclusive and equitable quality education” (SDG 4), highlighting the imperative of lifelong learning opportunities for all learners, including those with special educational needs (SEN) (UNESCO, 2016). In response, inclusive education aims to provide tailored support for SEN students, which is crucial not only for equity but also for fulfilling the core mission of public education in fostering literacy development (Slowík et al., 2022; Wakeman et al., 2021). However, students with SEN encounter persistent challenges in acquiring foundational academic skills, particularly in the domain of reading (Lemons et al., 2013; Lenkeit et al., 2022; Vargas et al., 2024; Virinkoski et al., 2022).

Reading fluency, defined as the ability to read connected texts accurately and quickly (Kim and Wagner, 2015), is a major predictor of reading comprehension (Chen et al., 2025; Hsu et al., 2023; Zhou et al., 2021). According to the direct and indirect effects model of reading (DIER; Kim, 2020), reading fluency itself is influenced by both word reading (e.g., orthographic knowledge) and listening comprehension (LC), and lower level skills like vocabulary have cascading indirect effects on reading fluency via upper level skills like LC. LC, the ability to understand spoken language, constitutes the foundational component of reading comprehension in the Simple View of Reading (SVR; Gough and Tunmer, 1986; Hoover and Gough, 1990).

Despite its theoretical importance, LC has been understudied in SEN research (Gacs et al., 2024). Existing literature has predominantly examined LC in typically developing primary school students (Kim, 2016, 2020; Lervåg et al., 2018; Memisevic et al., 2024), while research involving preschool children with special needs or those enrolled in specialized institutions remains scarce (Gao et al., 2024; Van Wingerden et al., 2018). Consequently, there is a gap in longitudinal research examining the developmental trajectory of LC among SEN students educated in mainstream inclusive classrooms. In addition, the results of previous studies on the factors influencing LC have been inconsistent (Bourdeaud'hui et al., 2020; Lin et al., 2015; Wolfgramm et al., 2016). Furthermore, while DIER posits these relationships, the specific contributions of LC and orthographic knowledge to reading fluency remain untested within the SEN population.

This study addresses these interconnected gaps within the context of Chinese inclusive education. The researchers explicitly acknowledge the inherent heterogeneity of the SEN population; the sample in this study, identified by teachers in mainstream schools, reflects the broad spectrum of SEN in real-world practice. While this approach limits our ability to draw conclusions about specific SEN subtypes, it provides an ecologically valid overview of a pressing practical issue. The primary aims of this longitudinal study are threefold: (1) to compare the level and one-year developmental trajectory of LC between pupils with SEN and their typically developing peers; (2) to identify key factors (e.g., vocabulary) influencing LC within the SEN group; and (3) to examine the joint contributions of LC and orthographic knowledge to reading fluency. By investigating these gaps, this research aims not only to test the applicability of the DIER framework in the heterogeneous SEN population but also to establish an evidence-based foundation for designing targeted interventions to support reading development among SEN students in inclusive settings.

## 2 Literature review

### 2.1 Listening comprehension in pupils with and without SEN

Listening comprehension (LC) refers to the capacity to understand spoken language, encompassing both the extraction of explicit information and the construction of meaning from context (Kim and Pilcher, 2016). As a higher-order cognitive skill, it demands the integration of various linguistic competencies, such as vocabulary and grammatical knowledge, alongside key cognitive functions, including working memory, inhibitory control, attention, reasoning, theory of mind, and comprehension monitoring (Florit et al., 2011; Gao et al., 2024; Kim, 2016, 2020; Kim and Pilcher, 2016). Serving as the foundation for reading comprehension development (Gough and Tunmer, 1986; Hoover and Gough, 1990), LC is widely recognized as critical to advancing reading proficiency and overall literacy outcomes. Despite its importance, research has predominantly examined LC in typically developing primary school students (Kim, 2016, 2020; Lervåg et al., 2018; Memisevic et al., 2024), leaving the developmental course and characteristics of LC in students with SEN largely underexplored.

Existing research on LC among children with special needs remains limited, with most studies focusing on preschool-aged children or students in specialized institutions rather than those with SEN in inclusive educational settings. Available evidence consistently indicates that these students experience significant delays in LC relative to typically developing peers. For example, preschoolers with autism spectrum disorder (ASD) demonstrated markedly poorer LC compared to age-matched controls (Gao et al., 2024). Van Wingerden et al. (2018) evaluated 76 students with intellectual disabilities attending special education schools and found substantial deficits compared to their typically developing counterparts. El-Wahed et al. (2024) examined 30 children aged 7 to 9 years and 11 months with reading difficulties, recruited from a hospital setting, and identified pronounced impairments in LC. LC underpins students’ ability to acquire and process new information, interpret teacher instructions, participate meaningfully in classroom discourse, and develop reading and writing proficiency (Marx et al., 2017; Wolfgramm et al., 2016). Therefore, it is essential to investigate the developmental trajectory of LC in SEN students and its influence on reading fluency to inform effective inclusive education practices.

Existing research on LC in pupils consistently identifies vocabulary as a key predictor, with students who possess more extensive vocabulary knowledge demonstrating superior performance in LC tasks (Bourdeaud'hui et al., 2020). For example, Wolfgramm et al. (2016), in a study of 354 sixth-grade pupils in Switzerland, found vocabulary to be the strongest predictor of LC. Kim (2016) reported that vocabulary exerted an indirect predictive effect on LC among first-grade pupils in South Korea. Lervåg et al. (2018), using six longitudinal assessments of 198 Norwegian second-grade pupils, confirmed vocabulary as a significant predictor of LC. Memisevic et al. (2024), in their evaluation of 77 Bosnian-speaking first- and second-grade pupils, identified vocabulary size as one of the most robust predictors of LC. Similarly, Xie et al. (2024), in a study of 167 third- and fourth-grade pupils learning English as a second language in China, demonstrated that vocabulary knowledge at Time 1 significantly predicted LC at Time 2.

In addition to vocabulary, research has examined gender as a potential factor influencing pupils’ LC; however, findings remain inconsistent. Some studies have found no significant gender differences in average LC scores among fourth- and sixth-grade students (Lin et al., 2015). Bourdeaud'hui et al. (2020, 2021) likewise reported no gender-based differences in LC among sixth graders. Notably, Wolfgramm et al. (2016) observed no general female advantage in LC among sixth graders, and, on one specific measure, found that male pupils performed significantly better than their female counterparts. These mixed results highlight the need for further research to clarify the role of gender in LC development, particularly among pupils with SEN, where such patterns remain underexplored.

Existing research has identified age as a critical factor influencing LC, with children’s abilities generally improving as they mature and acquire greater language experience. Lin et al. (2015) demonstrated that sixth-grade pupils scored significantly higher in LC compared to fourth-grade pupils, underscoring the developmental progression of this skill. Valentini and Serratrice (2023), through three longitudinal assessments of 100 bilingual pupils in the United Kingdom from first to second grade, observed steady improvements in LC across all participants over time. Similarly, Van Wingerden et al. (2018), in a three-year longitudinal study of 76 children with intellectual disabilities attending special education schools, reported gradual gains in LC, although these children consistently performed below their typically developing peers. Notably, the study did not clarify whether the gap in LC between children with intellectual disabilities and typically developing children widened or narrowed over time, leaving important questions about developmental trajectories unresolved.

In summary, current research on the factors influencing LC—such as vocabulary, gender, and age—has largely concentrated on typically developing students, with some findings remaining inconclusive. To date, no longitudinal study has systematically examined the combined effects of vocabulary, gender, and age on the development of LC in students with SEN, leaving a critical gap in the literature.

### 2.2 Reading fluency in pupils with SEN

Reading fluency refers to the cognitive process of accurately and rapidly decoding words while simultaneously directing attentional resources to text comprehension (Wolf and Katzir-Cohen, 2001). As a core academic skill, substantial empirical evidence identifies reading fluency as a major predictor of reading comprehension (Chen et al., 2025; Zhou et al., 2021). Deficits in reading fluency have broad negative effects, extending beyond language learning to other academic domains, undermining pupils’ motivation, limiting comprehension across subjects, and ultimately increasing the risk of academic failure. Research has consistently demonstrated that individual differences in reading fluency tend to widen over time, illustrating a Matthew effect pattern in reading development (Hui et al., 2018; Zhang et al., 2023). Therefore, investigating the developmental course of reading fluency in pupils with SEN and introducing early, targeted interventions holds both strong theoretical importance and clear practical relevance for enhancing reading comprehension and improving long-term academic outcomes.

Existing research on reading fluency in students with special needs has primarily concentrated on those with learning disabilities, particularly the dyslexia subtype, while giving limited attention to students with other disabilities. Reading fluency levels in these populations are closely linked to disability severity and consistently lag behind those of typically developing peers. For example, Holden-Pitt et al. (2008), in a longitudinal study of approximately 700 pupils with learning disabilities, found that although their oral reading fluency improved over 3 years, the study did not indicate whether the gap with typically developing peers narrowed or widened. Lemons et al. (2013), examining 7,440 students with significant cognitive disabilities—including intellectual disabilities, autism, learning disabilities, and other disabilities—in grades 3 to 8 and grade 11, reported that while passage oral reading fluency increased with grade level, it remained substantially below grade expectations, with students with learning disabilities outperforming those with intellectual or other disabilities. Wanzek et al. (2014) found that second-grade students with emotional disturbance or learning disabilities demonstrated significantly lower initial oral reading fluency compared to typically developing peers, with growth through third grade insufficient to close the gap. Solari et al. (2017) showed that students with autism spectrum disorder (mean age = 13.8 years) exhibited significantly poorer oral reading fluency than age-matched typically developing students. Zhang et al. (2024), comparing 81 Chinese students with dyslexia in grades 2, 4, and 6 to 81 age-matched controls, found that although the dyslexia group demonstrated significant grade-level gains in both silent sentence-level and oral passage-level reading fluency, their performance remained well below that of controls, with the silent reading fluency gap widening progressively across grades.

Existing research has largely focused on comparing reading fluency between students with SEN and typically developing peers, as well as on mapping the developmental trajectories of reading fluency within special populations, while giving comparatively little attention to the factors influencing reading fluency in these groups. Grounded in the Simple View of Reading, the DIER model posits that both lower-level skills and higher-order cognitive abilities are indirectly related to reading comprehension through multiple pathways (Kim, 2020). Within the DIER framework, LC and orthographic knowledge are identified as key predictors of reading fluency (Kim, 2020). However, research on reading fluency in special populations has not yet examined the predictive roles of either LC or orthographic knowledge. Notably, the mechanisms underlying reading comprehension in special populations may diverge from those observed in typically developing students, as highlighted by evidence showing that the Simple View of Reading does not fully account for reading comprehension in adolescents with intellectual disabilities (Nilsson et al., 2021). Determining whether LC and orthographic knowledge function as influential predictors of reading fluency in pupils with SEN thus remains an important area for future investigation.

### 2.3 Contributions of listening comprehension and orthographic knowledge on reading fluency

Existing research on the predictive role of LC in reading fluency remains limited, with most studies focusing on oral reading fluency rather than the silent reading fluency emphasized in the current study, and the findings have been inconsistent. Rooted in the Simple View of Reading, some studies suggest that reading comprehension results from the combined contributions of decoding and LC, with both components considered essential for successful reading comprehension (Hoover and Gough, 1990). This framework implies that LC begins to influence reading comprehension only after individuals reach a certain level of word reading proficiency. For example, Kim et al. (2012), in a two-year longitudinal study of 270 first-grade pupils, found that LC in first grade did not significantly predict silent reading fluency, but it emerged as a significant predictor in second grade. Kim et al. (2014), examining 98 Korean kindergarteners and 170 first-grade pupils, reported that LC significantly predicted oral reading fluency in first grade but not in kindergarten. Kim and Wagner (2015) showed that oral reading fluency partially mediated the relationship between LC and reading comprehension in English-speaking pupils from second to fourth grade. Metsala (2023) similarly found that LC significantly predicted oral reading fluency 18 months later among 83 English-speaking second and third graders. However, Kim et al. (2021), using six longitudinal assessments over 3 years with 371 first-grade English-speaking pupils, found that while LC in the fall of first grade significantly predicted oral reading fluency in the spring of first grade, LC in the fall of second grade no longer predicted oral reading fluency in the spring of second grade. These findings suggest that the predictive role of LC in reading fluency may diminish as pupils’ word reading skills develop, highlighting the need for further investigation into this relationship, particularly in diverse student populations.

Existing research has explored the correlation between LC and reading fluency in children with special needs, though its predictive role remains underexamined. Capin et al. (2021) assessed 446 fourth-grade American pupils performing below the 16th percentile on reading comprehension tests and identified a significant correlation between LC and silent reading fluency. Similarly, Solari et al. (2017) evaluated children with autism spectrum disorder (mean age = 13.8 years) and reported a significant correlation between LC and oral reading fluency. However, neither study extended its analysis to examine the predictive effect of LC on reading fluency, leaving a critical gap in understanding the developmental mechanisms linking these skills in special populations.

Reading fluency is typically assessed through measures of decoding accuracy and speed (Fuchs et al., 2001; Zhou et al., 2021). According to the Theory of Automatic Information Processing (LaBerge and Samuels, 1974), individuals possess limited cognitive resources; thus, the greater the cognitive effort devoted to word decoding, the fewer resources remain for comprehension, highlighting the role of orthographic knowledge as a key factor in reading fluency. The positive predictive effect of orthographic knowledge on reading fluency in alphabetic languages has been consistently demonstrated across multiple empirical studies involving pupils (Georgiou and Rothou, 2024; O’Brien et al., 2011; Papadopoulos et al., 2016; Rakhlin et al., 2019).

Approximately 82% of modern Chinese characters are compound characters composed of two core components: a semantic radical that conveys meaning and a phonetic radical that suggests pronunciation (Li et al., 2012). As a logographic writing system, Chinese differs fundamentally from alphabetic languages in its orthographic structure. Chinese orthographic knowledge involves the ability to infer pronunciation from phonetic radicals, extract meaning from semantic radicals, and apply structural rules governing character composition (Wang et al., 2014). Existing studies have consistently shown that orthographic knowledge significantly contributes to reading fluency in Chinese. For example, Tan et al. (2005), using pseudo-character copying tasks, found that writing skills were more strongly associated with reading fluency than phonological awareness. Liu et al. (2017), in a study of 1,776 pupils from grades 1 to 6 in Beijing, reported that orthographic knowledge significantly predicted reading accuracy, fluency, and silent reading speed, with these effects remaining consistent across varying reading ability levels. Hui et al. (2018), through three longitudinal assessments over 1 year with 177 first-grade pupils, demonstrated that orthographic knowledge predicted initial levels of reading fluency, with higher orthographic knowledge corresponding to stronger starting points. Similarly, Yu et al. (2023) found that first-grade pupils’ orthographic knowledge not only directly facilitated later reading fluency but also indirectly predicted it through character recognition. Wen et al. (2024), using longitudinal data from 149 first-grade pupils, observed significant positive correlations between early orthographic knowledge and subsequent reading fluency. In contrast, Zhang et al. (2021), examining 56 Chinese school-age children with hearing loss and age-matched peers with normal hearing, found that semantic competence—not orthographic knowledge—was the sole significant predictor of reading fluency in both groups. These findings suggest that the relationship between orthographic knowledge and reading fluency may differ for Chinese children with special needs, highlighting the need for further investigation in this population.

### 2.4 Present study

Enhancing reading comprehension skills in primary school students with SEN presents a significant challenge in the pursuit of high-quality inclusive education. The present study had three primary objectives. First, it aimed to compare the one-year developmental trajectory of LC between pupils with SEN and their typically developing peers. Based on evidence of persistent language-processing challenges in SEN populations, we hypothesized that pupils with SEN would exhibit lower baseline LC and that this performance gap would widen over time. Second, the study sought to identify the key predictors of LC within the SEN group using robust regression analysis. Aligning with prior research identifying vocabulary as a foundational language skill essential for LC (Kim, 2016; Memisevic et al., 2024; Xie et al., 2024), the researchers hypothesized that vocabulary would be a significant positive predictor of LC, whereas demographic factors (gender and grade) would not. Third, guided by the DIER model (Kim, 2020), the study aimed to test the hypothesis that reading fluency is directly predicted by both LC and orthographic knowledge. By examining these relationships, the researchers evaluated the applicability of the DIER framework in a heterogeneous SEN sample and aims to identify key leverage points for designing targeted instructional strategies to enhance LC and reading fluency in inclusive classrooms.

## 3 Method

### 3.1 Participants

Participants were recruited from two mainstream elementary schools in China. Pupils with SEN were initially identified by their class teachers and subject instructors following the school’s established SEN identification protocol: (1) persistent academic underperformance, ranking in the bottom 10% of the class academically, and (2) behavioral markers based only on teacher reports (e.g., attention deficits, social communication difficulties). This approach yields a pedagogically defined, heterogeneous sample of students experiencing significant learning and behavioral difficulties in mainstream settings, thereby reflecting the practical realities of inclusive education. Following this preliminary screening, the research team was invited by the schools to carry out comprehensive assessments of the identified students. These evaluations were conducted in quiet, unoccupied classrooms on school premises to ensure standardized testing conditions and minimize potential distractions. Researchers confirmed that this study was conducted in accordance with the relevant ethical standards, and parental consent was obtained through head teachers.

The researchers explicitly acknowledge the heterogeneity of this teacher-identified sample, which was reflected in its demographic composition, the spectrum of teacher-reported concerns, and the limited prevalence of formal certification (Bruggink et al., 2013). The final sample consisted of 103 pupils with SEN, including 57 from School A and 46 from School B. Participants were distributed across grades 1–6, with 24 in Grade 1, 25 in Grade 2, 23 in Grade 3, 12 in Grade 4, 12 in Grade 5, and 7 in Grade 6. A gender imbalance was observed (21 female, 82 male), which aligns with the reported higher prevalence of having SEN among boys (Daniel and Wang, 2023; Keating et al., 2025). For a subset of 48 pupils from School A, teacher-supplied remarks illustrated the range of presenting concerns, including concerns related to attention-deficit/hyperactivity behaviors (*n* = 21), intellectual disability (*n* = 10), autism spectrum behaviors (*n* = 9), learning disabilities (*n* = 7), and other developmental delays. Among these 103 students, 17 held official disability certificates, 72 lacked such documentation, and 14 did not report their certification status.

### 3.2 Measures

#### 3.2.1 Listening comprehension

LC was evaluated using the Children’s Oral Language Comprehension Test (COLCT) developed by Lin and Chi (1999), which assesses four core domains: listening memory, grammatical comprehension, semantic judgment, and discourse comprehension. Listening memory refers to the ability to temporarily retain language information in short-term memory and apply working memory processes to follow instructions. Grammatical comprehension reflects the understanding of Chinese grammatical structures, including word order, passive constructions, question forms, temporal adverbs, pronouns, adjective and adjective combinations, conjunctions, and complex sentences. Semantic judgment assesses the ability to detect semantically incorrect words or sentences and provide appropriate corrections. Discourse comprehension evaluates the capacity to understand main ideas, recall factual details, and draw inferences (Chi, 2007). The test includes 18 items on listening memory, 24 on grammatical comprehension, 26 on semantic judgment, and 32 on discourse comprehension, with the total raw score calculated as the sum of all subtest scores. The interviewer orally presented each question stem once, and the child responded after listening. Each item was scored 0 for an incorrect response and 1 for a correct response. For example, the interviewer orally presented the phrase “I eat with my ears,” and the child was expected to identify “ears” as a semantically incorrect word for “eat” and instead respond with “I eat with my mouth” or another appropriate answer. The Children’s Oral Language Comprehension Test has demonstrated strong internal consistency, content validity, and test–retest reliability (Chi, 2007; Lin and Chi, 1999). In this study, Cronbach’s alpha coefficients for the pretest and posttest in Schools B and A were 0.876, 0.900, and 0.948, respectively.

#### 3.2.2 Vocabulary

The Chinese version of the Peabody Picture Vocabulary Test-Revised (Sang and Miao, 1990), adapted from the original Peabody Picture Vocabulary Test-Revised (Dunn and Dunn, 1981), was employed to assess vocabulary skills among pupils with SEN. In Sang and Miao’s validation study, the split-half reliability and test–retest reliability coefficients were reported as 0.99 and 0.938, respectively, indicating strong reliability. In the present study, pupils with SEN were instructed to select the picture that best corresponded to a word spoken aloud by the evaluator from a set of four images. The assessment was discontinued if a participant made six errors within any sequence of eight consecutive items.

#### 3.2.3 Reading fluency

A three-minute reading task developed by Lei et al. (2011) was employed to assess the reading fluency of pupils with SEN. The task consisted of 100 simple sentences, generally arranged in order of increasing length. During the assessment, children were instructed to silently read each sentence as quickly as possible within the three-minute time limit and determine whether the sentence was correct (e.g., “The sun gives us light and heat (√)” vs. “Many people live on the moon (×)”). The final score was calculated by subtracting the total number of Chinese characters in incorrectly judged sentences from the total number of characters in correctly judged sentences, with higher scores reflecting greater reading fluency. In this study, the Cronbach’s alpha coefficient for the reading fluency task was 0.927, indicating high internal consistency.

#### 3.2.4 Orthographic knowledge

The Orthographic Knowledge Test, adapted by Wang et al. (2014), was used to evaluate orthographic knowledge in pupils with SEN. The assessment included two subtests: a reversed-character judgment task and a pseudo-character discrimination task, each containing 40 items. Each item was scored as 1 point, yielding a total possible score of 80 points. This test has been validated in Chinese children with dyslexia (Wang et al., 2014). In the present study, the test demonstrated high internal consistency, with a Cronbach’s alpha coefficient of 0.936.

### 3.3 Test procedure and statistical analyses

All evaluators were undergraduate students majoring in special education who received professional training. Specifically, they completed an intensive training program supervised by a senior special education specialist with a doctoral degree, and they were required to pass a final competency assessment (achieving ≥90% accuracy on mock evaluations) prior to data collection. Reading fluency and orthographic knowledge assessments were administered in group settings within quiet classrooms; while LC and vocabulary evaluations were conducted individually by evaluators in separate rooms to ensure standardized administration. All 103 SEN pupils completed the baseline assessment (Time 1): School A in December 2024 and School B in December 2023. A follow-up assessment of LC was conducted for the School B cohort between November and December 2024. During this period, 4 pupils were lost to attrition due to school transition, resulting in a longitudinal sample of 42 SEN pupils for analysis. Data analyses were carried out using the stats, psych, dplyr, rstatix and robustbase packages in R software (version 4.5.0).

## 4 Results

### 4.1 Preliminary analysis

Table 1 presents the descriptive statistics for the key variables assessed in the study. The baseline data for pupils with SEN were established by combining the wave 1 data from School A and School B assessed using standardized tools. To evaluate the grade-norm-referenced performance of pupils with SEN in LC, raw scores were converted to T-scores (*M* = 50, SD = 10) following Lin and Chi’s (1999) standardization guidelines. As indicated in Table 1, pupils with SEN mean LC T-score fell within the “below average” range (*M* = 36.50).

**Table 1:** Descriptive statistics for vocabulary, listening comprehension, orthographic knowledge, and reading fluency in pupils with SEN.

Variables	*N*	*M*	SD	Range
Vocabulary	94	94.74	43.85	0–168
Listening memory	94	7.88	4.93	0–18
Grammatical comprehension	94	10.12	7.56	0–24
Semantic judgment	94	7.96	8.62	0–26
Discourse comprehension	94	12.02	9.77	0–31
Listening comprehension raw score	94	37.98	29.25	0–94
Listening comprehension T-score	94	36.50	16.30	4–71
Orthographic knowledge	37	59.70	15.28	28–77
Reading fluency	38	294.68	306.93	−96–920

*N* = sample size; *M* = mean; SD = standard deviation. According to Lin and Chi’s (1999) classification, *T*-scores above 70 were designated as “exceptionally superior,” 60–70 as “superior,” 40–60 as “average,” 30–40 as “below average,” and below 30 as “significantly below average”.

### 4.2 Longitudinal changes and regression explaining listening comprehension in SEN pupils

A one-year follow-up assessment of LC was conducted with School B pupils, with 4 sixth-grade students lost to attrition due to transitioning to secondary school, resulting in a final sample of 42 participants. The Shapiro–Wilk test revealed non-normally distributed scores for pretest grammatical comprehension (*W* = 0.906, *p* = 0.002), pretest semantic judgment (*W* = 0.795, *p* < 0.001), pretest discourse comprehension (*W* = 0.894, *p* = 0.001), pretest LC raw score (*W* = 0.944, *p* = 0.040), pretest LC T-score (*W* = 0.926, *p* = 0.010), posttest listening memory (*W* = 0.946, *p* = 0.047), posttest semantic judgment (*W* = 0.884, *p* < 0.001), and posttest discourse comprehension (*W* = 0.934, *p* = 0.018), while pretest listening memory (*W* = 0.958, *p* = 0.123), posttest grammatical comprehension (*W* = 0.972, *p* = 0.378), posttest LC raw score (*W* = 0.981, *p* = 0.687), and posttest LC T-score (*W* = 0.964, *p* = 0.197) were normally distributed. These findings justified the use of Wilcoxon signed-rank tests in subsequent statistical testing.

Wilcoxon signed-rank tests were performed to compare pupils with SEN LC performance between the pretest and posttest. Results indicate that pupils with SEN pretest scores were significantly lower than posttest scores across listening memory (*Z* = −5.305, *r* = 0.819), grammatical comprehension (*Z* = −4.814, *r* = 0.743), semantic judgment (*Z* = −4.504, *r* = 0.695), discourse comprehension (*Z* = −5.020, *r* = 0.775), and overall LC raw score (*Z* = −5.453, *r* = 0.841) (all *p* < 0.001). However, the Wilcoxon signed-rank test showed a marginal decline in posttest LC T-scores (*Z* = −1.852, *p* = 0.064, *r* = 0.286). It revealed that despite within-group improvements, pupils with SEN LC continued to fall further behind grade-level expectations, indicating a widening performance gap relative to their typically developing peers (see Table 2).

**Table 2:** Performances of pupils with SEN on listening comprehension between the pre-test and post-test (*N* = 42).

Variables	Pre-test		Post-test		Z	*p*	r
	*M* ± SD	Mdn (Q1, Q3)	*M* ± SD	Mdn (Q1, Q3)			
LM	5.55 ± 3.28	6.00 (3.25, 7.75)	8.55 ± 3.74	9.00 (7.00, 11.00)	−5.305^***^	<0.001	0.819
GC	6.57 ± 5.22	6.50 (0.25, 10.00)	9.88 ± 4.45	10.00 (7.25, 13.00)	−4.814^***^	<0.001	0.743
SJ	2.81 ± 3.44	1.50 (0.00, 4.75)	4.95 ± 4.80	4.00 (0.25, 7.00)	−4.504^***^	<0.001	0.695
DC	6.14 ± 5.62	4.50 (0.00, 10.00)	8.74 ± 6.65	8.00 (3.25, 14.00)	−5.020^***^	<0.001	0.775
LCRS	21.07 ± 15.55	24.00 (7.00, 30.00)	32.12 ± 17.62	31.50 (22.00, 42.75)	−5.453^***^	<0.001	0.841
LCTS	27.62 ± 7.54	27.50 (21.00, 34.75)	25.19 ± 11.02	26.50 (20.25, 31.00)	−1.852^~^	0.064	0.286

*M* = mean; SD = standard deviation; Mdn (Q1, Q3) = median (interquartile range); Z = Wilcoxon signed-rank test statistic; ~*p* < 0.10, *p < 0.05, **p < 0.01, ***p < 0.001; r = effect size; LM = listening memory; GC = grammatical comprehension; SJ = semantic judgment; DC = discourse comprehension; LCRS = listening comprehension raw score; LCTS = listening comprehension T-score.

To investigate the effects of gender, grade, and vocabulary on pupils with SEN’s LC, Shapiro–Wilk tests were first conducted to assess the normality of the data distributions. The results revealed non-normal distributions for both LC (*W* = 0.921, *p* < 0.001) and vocabulary (*W* = 0.949, *p* = 0.001). Consequently, a robust regression analysis was conducted using R 4.5.0, with gender, grade, and vocabulary entered as independent variables and LC as the dependent variable.

As depicted in Table 3, vocabulary demonstrated a significant positive effect on LC (*β* = 0.74, 95% CI [0.58, 0.90]), with an effect size substantially larger than other variables. Specifically, for every one standard deviation increase in vocabulary scores, LC scores increased significantly by 0.74 standard deviations. Gender differences did not reach statistical significance (*β* = −0.25, 95% CI [−0.54, 0.05]), though the effect size suggested a marginal advantage for male students. None of the grades showed statistically significant effects, as all confidence intervals included zero.

**Table 3:** Robust regression of listening comprehension in pupils with SEN (*N* = 94).

Variables	Std. Coef.	95% CI	*p*
Intercept	−0.03	[−0.32, 0.26]	
Vocabulary	0.74	[0.58, 0.90]	<0.001
Gender (Ref: Male)	−0.25	[−0.54, 0.05]	0.104
Grade (Ref: Grade 1)			
-Grade 2	−0.20	[−0.73, 0.33]	0.452
-Grade 3	0.20	[−0.18, 0.58]	0.297
-Grade 4	0.35	[−0.04, 0.73]	0.075
-Grade 5	0.36	[−0.12, 0.84]	0.139
-Grade 6	−0.19	[−0.90, 0.52]	0.598

Std. Coef. = standardized coefficient; CI = confidence interval; Ref = reference group.

### 4.3 Regression explaining reading fluency among Chinese pupils with SEN

As depicted in Table 4, vocabulary, LC, orthographic knowledge, and reading fluency were all significantly interrelated, with correlation coefficients ranging from 0.37 to 0.70. All correlation tests were corrected for multiple comparisons using the Benjamini-Hochberg FDR method (q < 0.05), including the weak correlation between LC and orthographic knowledge (Rho = 0.370, *p* = 0.029). These results indicate strong associations between the core language and reading skills in pupils with SEN. The findings highlight the importance of considering multiple interacting factors when examining reading fluency development in this population.

**Table 4:** Intercorrelations among variables used in the study (*N* = 35).

Variables	*M* ± SD	1	2	3
1 Vocabulary	114.03 ± 40.82	1		
2 Listening comprehension raw score	61.77 ± 26.64	0.62^***^		
3 Orthographic knowledge	59.86 ± 15.48	0.57^***^	0.37^*^	
4 Reading fluency	324.09 ± 301.78	0.52^**^	0.70^***^	0.59^***^

*M* = mean; SD = standard deviation. *p < 0.05, **p < 0.01, ***p < 0.001.

The Shapiro–Wilk test was first conducted to assess the normality of the distributions for vocabulary, LC, orthographic knowledge, and reading fluency in pupils with SEN. The results indicated non-normal distributions across all variables: vocabulary (*W* = 0.887, *p* = 0.002), LC (*W* = 0.916, *p* = 0.011), orthographic knowledge (*W* = 0.828, *p* < 0.001), and reading fluency (*W* = 0.933, *p* = 0.034). These findings justified the use of robust regression analyses in subsequent statistical testing.

Using R 4.5.0, a robust regression analysis was conducted with vocabulary, LC, and orthographic knowledge as predictors and reading fluency as the outcome variable. As shown in Table 5, both LC (*β* = 0.57, 95% CI [0.26, 0.89]) and orthographic knowledge (*β* = 0.33, 95% CI [0.11, 0.56]) demonstrated significant positive effects on reading fluency. Together, these three predictors accounted for 53.9% of the variance in reading fluency among pupils with SEN.

**Table 5:** Robust regression analysis of reading fluency in pupils with SEN (*N* = 35).

Variables	Std. Coef.	95%CI	*p*
Intercept	0.01	[−0.26, 0.28]	
Vocabulary	−0.05	[−0.42, 0.32]	0.774
Listening comprehension raw score	0.57	[0.26, 0.89]	<0.001
Orthographic knowledge	0.33	[0.11, 0.56]	0.005

Std. Coef. = standardized coefficient; CI = confidence interval.

## 5 Discussion

This longitudinal study investigated the development of LC and its role in reading fluency among a heterogeneous group of pupils with SEN in inclusive Chinese primary schools. Guided by the DIER model, researchers in this study found (1) pupils with SEN exhibited lower LC than their typically developing peers, and this gap widened over 1 year; (2) vocabulary was a strong, positive predictor of LC within the SEN group, whereas demographic variables (gender, grade) were not when vocabulary was accounted for (3) both LC and orthographic knowledge predicted reading fluency.

### 5.1 Divergence in listening comprehension between pupils with and without SEN

Our study confirms a persistent and expanding delay in LC among pupils with SEN. This finding aligns with prior research on specific subgroups, such as children with intellectual disabilities or dyslexia (El-Wahed et al., 2024; Van Wingerden et al., 2018). According to the DIER model, LC is a higher-order skill that draws upon foundational language abilities (e.g., vocabulary, syntax) and cognitive components (e.g., working memory, reasoning; Kim, 2016, 2020). The significant heterogeneity within our SEN sample implies that different students likely have vulnerabilities in different components of this complex system. Some may struggle with deficits in executive functioning (Di Lieto et al., 2020) or lexical and cognitive skills (Kesäläinen et al., 2022), while others face core deficits in auditory filtering of irrelevant stimuli (Armstrong-Gallegos and Nicolson, 2020). Consequently, our findings represent the aggregate average disadvantage in LC for this mixed population.

Longitudinally, the decline in standardized T-scores despite improvement in raw scores indicates that the developmental rate of LC in pupils with SEN lags behind normative expectations. These findings extend the work of Van Wingerden et al. (2018), who did not examine changes in the LC gap over time. This divergence may be explained not only by the inherent linguistic and cognitive deficits underlying LC in pupils with SEN but also by comparative social mechanisms. Specifically, pupils with SEN enrolled in mainstream schools experience stronger “big-fish-little-pond effects,” as upward social comparisons diminish perceptions of academic competence, thereby undermining academic self-concept and learning motivation (Lohbeck et al., 2024). Moreover, the tendency to evaluate academic performance more negatively (Bruggink et al., 2013) creates a self-reinforcing cycle that aligns with a Matthew effect, in which the LC gap between pupils with SEN and typically developing peers progressively widens with age.

### 5.2 Vocabulary as the core predictor of listening comprehension in pupils with SEN

Vocabulary emerged as the strongest predictor of LC among pupils with SEN, supporting the DIER model’s conceptualization of vocabulary as a foundational language skill for comprehension (Kim, 2020). LC involves a series of complex cognitive operations, including separating speech from background noise, parsing the speech stream to identify word boundaries, selecting appropriate lexical items from multiple activated candidates, and integrating these words into broader sentence-level meaning (Andringa et al., 2012). A high proportion of unfamiliar vocabulary can create lexical gaps that disrupt this process (Bourdeaud'hui et al., 2020), and this challenge is further intensified by the transient nature of spoken input, where missed words can result in comprehension failures due to the real-time processing demands of listening (Hagtvet, 2003).

The study revealed that vocabulary had a significant positive effect on LC, whereas gender and grade did not show predictive influence. It is hypothesized that the strong impact of vocabulary may have masked potential effects of gender and grade. To examine this possibility, an alternative model was tested excluding vocabulary as a predictor. This modified model revealed the expected patterns: both gender and grade emerged as significant predictors, with girls performing significantly lower than boys (*β* = −0.50, *p* = 0.011), and pupils in grades 2–4 outperforming first graders (Grade 2: *β* = 0.68, *p* = 0.002; Grade 3: *β* = 1.07, *p* < 0.001; Grade 4: *β* = 1.52, *p* < 0.001). The model explained 27.9% of the variance in LC scores (*R*^2^ = 0.279). The contrast between the two models highlights the central role of vocabulary in explaining LC variance within this population.

The finding that boys outperformed girls in this alternative model may reflect a referral bias in teacher-based SEN identification. The SEN identification process encompasses not only objective assessments of academic skills but also teachers’ subjective judgments regarding behavioral and social–emotional factors, which are significant predictors of SEN classification (Smeets and Roeleveld, 2016). Externalizing behaviors such as hyperactivity, impulsivity, and aggression, which are more prevalent among boys (Van Der Veen et al., 2010), may lead to the classification of male pupils with adequate LC but behavioral challenges as SEN cases. In contrast, girls with fewer externalizing behaviors may be under-identified unless they exhibit pronounced LC deficits. This pattern, consistent with broader findings that boys are more likely to be classified as having SEN (Bruggink et al., 2013; Daniel and Wang, 2023; Keating et al., 2025), underscores that our sample’s heterogeneity is shaped by real-world educational practices.

### 5.3 Listening comprehension and orthographic knowledge as predictors of reading fluency in pupils with SEN

Our finding that both LC and orthographic knowledge predicted reading fluency provides empirical support for the application of the DIER model to SEN populations. The DIER model integrates the core tenets of the Simple View of Reading (Hoover and Gough, 1990) while specifying the network of direct and indirect effects from lower level skills like vocabulary to upper level skills like LC and orthographic knowledge (Kim, 2020).

First, the predictive role of LC aligns with previous research on typically developing pupils and teenagers (Bar-Kochva et al., 2023; Kim et al., 2012, 2014, 2021; Metsala, 2023). This relationship likely arises because the core cognitive skills required for LC (e.g., working memory and attention), basic language competencies (e.g., vocabulary, grammatical knowledge), and higher-order cognitive abilities (e.g., reasoning, theory of mind, and comprehension monitoring) (Kim, 2016) are also fundamental to reading fluency development.

Second, orthographic knowledge significantly predicts reading fluency, consistent with studies on typically developing Chinese pupils (Hui et al., 2018; Liu et al., 2017; Wen et al., 2024; Yu et al., 2023). The reading fluency task used in the present study required rapid sentence judgment and semantic processing, which is particularly relevant in the context of Chinese, where character recognition relies heavily on direct visual-to-semantic mapping (Zhou et al., 1998). Orthographic knowledge, defined as the understanding of character components (semantic and phonetic radicals) and structural rules (Wang et al., 2014), likely facilitates this mapping process (Yu et al., 2023). For pupils with SEN, stronger orthographic knowledge enables more efficient and accurate character recognition, thereby enhancing both the precision and automaticity essential for fluent reading.

Notably, vocabulary did not exert a direct effect on reading fluency in the final model. This suggests that for pupils with SEN, vocabulary’s influence on reading fluency may be primarily mediated through LC and orthographic knowledge. As a lower level skill, vocabulary underpins both LC and orthographic knowledge (Kim, 2020), thus, its direct predictive effect may be overshadowed by these more proximal factors. This interpretation is supported by vocabulary’s prediction of LC, as well as the positive correlation between vocabulary and orthographic knowledge. Pupils with richer vocabularies develop more refined mental lexicon representations, facilitating faster semantic access (Schmitt, 2014); this mechanism theoretically supports reading fluency, although it was not captured as a direct effect in our model.

## 6 Implications

These findings provide important empirical support for developing targeted interventions to strengthen LC and reading fluency in pupils with SEN. First, given the persistent LC difficulties observed in pupils with SEN, schools should adopt multifaceted interventions aimed at enhancing this skill to support broader gains in reading comprehension. Prior research demonstrates that LC is responsive to intervention, as affirmed by Solari et al.’s (2020) storybook-based program for pupils with ASD, which improved both listening and reading comprehension outcomes. Second, the significant positive effect of vocabulary on LC suggests that strengthening vocabulary could enhance listening outcomes. Vocabulary in pupils with SEN is highly amenable to intervention, as demonstrated by Eden and Shmila’s (2023) study using the Osmo™ app—a multisensory hybrid learning technology—which significantly improved vocabulary scores in pupils with dyslexia. Third, the identified positive impact of orthographic knowledge on reading fluency highlights the potential benefits of orthographic training for pupils with SEN. Lin’s (2021) intervention with third-grade Chinese pupils with dyslexia showed that comprehensive orthography-based instructional packages improved both orthographic knowledge and reading fluency. Fourth, implementing evidence-based reading fluency interventions is crucial. Studies such as that of Martins et al. (2023) demonstrate the efficacy of adapted reading fluency programs (i.e., HELPS-PB). Building on this evidence, adapting or developing such targeted programs for Chinese SEN students could directly improve their reading fluency.

These findings also offer policymakers critical guidance for improving inclusive education strategies. First, although 1 year of inclusive education led to measurable gains in LC among pupils with SEN, their grade-norm-referenced T-scores exhibited a marginal but significant posttest decline, indicating a widening gap relative to typically developing peers. This finding suggests that mainstream placement alone is insufficient to meet the specific needs of pupils with SEN and underscores the necessity of implementing more intensive LC interventions (Fuchs et al., 2018) to close this gap. Second, the SEN identification rate of 1% across the participating schools was notably below the 7–30% range reported in prior research (Keating et al., 2025; Kesäläinen et al., 2022; Van Der Veen et al., 2010), with male pupils outnumbering females at a ratio of 3.90:1—considerably higher than the 1.38–2.57:1 ratios reported in previous studies (Kesäläinen et al., 2022; Van Der Veen et al., 2010)—indicating likely underidentification, particularly of female pupils. In developing countries, this gap likely stems from multiple factors, including limited educational resources per student (e.g., limited teacher training on SEN screening). To address this gap, inclusive education administrators should improve teacher-based identification procedures to ensure that more pupils with SEN, especially girls, are appropriately identified and supported.

## 7 Limitations

The primary limitation is the heterogeneity of the SEN sample. Although the researchers have detailed this diversity and focused on teacher-identified students with significant academic and behavioral difficulties, aggregating across different SEN subtypes limits insights into the linguistic profiles and developmental trajectories of specific subgroups. The present research findings should be interpreted as describing average tendencies for this pedagogically defined group. Future research must recruit larger, clinically characterized subsamples to test the model’s specificity and generalizability.

Additional limitations are as follows. First, the sample was drawn from only two elementary schools in a single Chinese city, limiting the generalizability of the findings to other educational settings and requiring cautious interpretation. Second, the study did not account for the role of vocabulary depth, despite evidence from bilingual school-aged children suggesting that vocabulary depth, rather than breadth, is a significant predictor of LC (Valentini and Serratrice, 2023)—an important distinction for future research to consider. Third, the assessment focused exclusively on silent reading fluency, leaving oral reading fluency unexamined; subsequent studies should distinguish between these reading modalities. Fourth, the two-wave longitudinal design provided limited data to characterize the developmental trajectories of LC in pupils with SEN, highlighting the need for extended longitudinal tracking to better understand the mechanisms underlying these skills. Lastly, contextual factors such as home and school environments, student motivation, and school engagement—factors known to influence the development of LC and reading fluency—were not measured; future research should incorporate these variables (e.g., teacher ratings and attendance) to more comprehensively investigate the determinants of language development in SEN populations.

## Data Availability

The original contributions presented in the study are included in the article/supplementary material, further inquiries can be directed to the corresponding author.
